# Assessment of hospital length of stay and direct costs of type 2 diabetes in Hubei Province, China

**DOI:** 10.1186/s12913-017-2140-4

**Published:** 2017-03-14

**Authors:** Dajie Chen, Shuai Liu, Xiaodong Tan, Qihan Zhao

**Affiliations:** Wuhan University, 115# Donghu Road, Wuhan, 430071 China

**Keywords:** Type 2 diabetes mellitus, Length of stay, Direct costs, Admission characteristics, Complications

## Abstract

**Background:**

The incidence of type 2 diabetes is increasing, creating a huge burden for China’s social healthcare system. This study aimed to evaluate hospital length of stay (LOS) based on admission characteristics and direct costs correlated with various types of complications for type 2 diabetic inpatients in Hubei Province, China.

**Methods:**

A total of 1528 inpatients diagnosed with type 2 diabetes discharged between April 1, 2013, and March 31, 2014, were included in this study. Information regarding patients’ admission and hospitalization were obtained from the hospital information system. The relationship between admission characteristics and LOS, distribution of total costs, and types of complications were described and analysed.

**Results:**

(1) The mean LOS was 11.65 days (median: 10 days). Multiple linear regression analysis demonstrated that inpatients with New Cooperative Medical Scheme (NCMS), aged 80 and above, had longer LOS than the reference group, and inpatients with chronic or acute + chronic complications had shorter LOS than those without. (2) Mean total costs per patient were US$159.72 ± 130.83 (median: US$135.33), US$240.60 ± 166.58 (median: US$192.09), and US$247.98 ± 166.22 (median: US$200.99) for inpatients with no complications, chronic complications, and acute + chronic complications, respectively. Total and individual costs were significantly less for patients without complications than for those with the two types of complications (*p* < 0.001). (3) Mean total costs per patient were US$225.40 ± 115.32 (median: US$200.34), US$221.25 ± 177.64 (median: US$170.05), and US$275.18 ± 193.14 (median: US$217.91) for inpatients with microvascular complications, macrovascular complications, and microvascular + macrovascular complications, respectively. Total costs were significantly higher for patients with microvascular + macrovascular complications than for those with other types of chronic complications (*p* < 0.001). (4) Drugs were the greatest expense for patients, and the least expensive treatment was nursing care.

**Conclusions:**

Medical insurance status, age, and type of complication may help to predict LOS for patients with type 2 diabetes in Hubei Province, China. The total and individual costs for patients with complications were higher than for those without, and hospitalization expenses posed a heavy burden. Efforts should be made to reduce the financial impact on patients by integrating the medical insurance system of urban and rural areas, and by reducing the risk of complications, especially microvascular complications.

**Electronic supplementary material:**

The online version of this article (doi:10.1186/s12913-017-2140-4) contains supplementary material, which is available to authorized users.

## Background

With global industrialization, urbanization, and modernization, the number of people afflicted with type 2 diabetes is growing year by year, with a projected increase from current estimates of 240 million, 6% of adults, affected to some 380 million, or 7% of adults, by 2025 [1]. In developing countries, a low level of health literacy and rapid economic growth are expected to continue to lead to higher prevalence of the disease. According to data from the International Diabetes Federation, between 2010 and 2030, there will be a 69% increase in numbers of adults with diabetes in developing countries, with 36% of the anticipated total global increase of 154 million people with diabetes projected to occur in India and China alone [2].

In recent years, huge changes have been taking place in Chinese society in terms of lifestyle and diet [3, 4]. Increase in physical inactivity, unhealthy eating habits, obesity, and smoking have led to widespread incidence of diabetes [5, 6]. According to an epidemiological study of diabetes in China, the age-standardized prevalence of total diabetes and prediabetes were 9.7 and 15.5%, respectively, accounting for 92.4 million adults with diabetes and 148.2 million adults with prediabetes [7].

In China, the treatment of patients with diabetes has created significant problems for the social healthcare system. A systematic review of the direct economic burden of type 2 diabetes in China showed that the direct medical cost increased from US$250 million in 1993 to US$8.65 billion in 2008. The annual total inpatient cost increased from US$30 million in 1993 to US$3.13 billion in 2007, with an average annual growth rate of 19.90%, which was more than the average annual growth rate of the gross domestic product (GDP (12.77%) and national health expenditures (12.88%) from 1993 to 2003 [8].

Inpatient costs were a substantial portion of health expenditures and were comprised of both direct and indirect costs; direct costs included cost of diagnosis and treatment of diabetes and its complications, and indirect costs represented productive losses resulting from prolonged length of stay (LOS), temporary or permanent disability, and premature death. This has been the subject of much research globally, but few studies have been carried out in Hubei Province, China. Therefore, assessing diabetic inpatients’ LOS and hospitalization expenses to reflect the patients’ burden was both necessary and urgent. This study aimed to estimate the LOS and direct costs of inpatients by analysing data on admission characteristics and a variety of complications and provide some suggestions for reducing the financial burden associated with type 2 diabetes.

A number of previous studies of LOS and direct costs of type 2 diabetes and its complications have been made [9, 10]. However, they failed to relate to the content of these two aspects simultaneously [11, 12] and offered little analysis of direct costs with regard to the various types of complications in type 2 diabetes. The most prominent feature of this study was that the information of direct costs was explored from the perspectives of no complications, chronic complications (microvascular complications, macrovascular complications, and microvascular + macrovascular complications), acute complications, and acute + chronic complications.

## Methods

### Data source and sample inclusion/exclusion criteria

The study was carried out in Zhongnan Hospital, a large tertiary general hospital located in the capital of Hubei Province, China. Data on inpatients between April 1, 2013, and March 31, 2014, whose principal discharge diagnosis was type 2 diabetes were obtained from the hospital information system (HIS), with diagnosis of type 2 diabetes determined in accordance with World Health Organization guidelines [13]. Criteria for inclusion in this study were the following: (1) both the main discharge diagnosis and secondary diagnosis were type 2 diabetes or its complications, or the main discharge diagnosis ICD-10 coding was E11; (2) age ≥18 years. Exclusion criteria were the following: (1) gestational diabetes; (2) neonatal diabetes; (3) transfer treatment; (4) deficits with primary data; (5) non-diabetic complications such as malignant tumours, fractures, and gastric ulcers. For inpatients undergoing two or more hospitalizations, each hospitalization was studied as a separate case, with our research focused on diabetic cases. The analysis ultimately consisted of 1528 cases.

### Data collection, study parameters, and definitions

Inpatients’ medical record information was exported to Microsoft Excel from the HIS (Additional file 1) and included (1) basic information such as hospitalization number, medical record number, type of medical insurance, name, gender, age, occupation, method of admission, admission time, discharge time, LOS, admission diagnosis, main discharge diagnosis, and other discharge diagnosis; (2) cost information for drugs, accommodation, imaging, therapeutics, laboratory analysis, sanitary materials, diagnostics, and nursing care. Information in Excel was preliminarily selected according to the inclusion and exclusion criteria and the above-mentioned information was preserved. Setting the year 2013 as the standard, the hospitalization costs were adjusted using China’s consumer price index in order to take into account the influence of inflation on health care costs. Inpatients were classified into four groups according to the type of complications: no complications, chronic complications, acute complications, and acute + chronic complications. Chronic complications were further subdivided into three categories: microvascular complications, macrovascular complications, and microvascular + macrovascular complications. Diabetes related complications included in the analysis were identified based on the ICD-10 codes of complications [14] and classified as either (1) acute, including coma with or without ketoacidosis, hyperosmolar coma, hypoglycaemic coma, hyperglycaemic coma, acidosis, and ketoacidosis (http://apps.who.int/classifications/icd10/browse/2010/en#/E10–E14), or (2) chronic, including macrovascular complications (acute myocardial infarction, angina pectoris, stroke, and non-traumatic amputation and foot ulcers) and microvascular complications (various levels of nephropathy [i.e., microalbuminuria, clinical proteinuria and chronic renal failure], retinopathy [i.e., background and proliferative retinopathy, macular edema, cataracts, and blindness], and peripheral neuropathy [11]).

### Statistical analysis

Numeric variables were reported as Mean ± standard deviation (SD), Median/25th–75th percentile, and categorical variables were described as both numbers and percentages.

Because LOS and hospitalization costs did not conform to the normal distribution, the Mann–Whitney *U* test (with 2 subgroups) and Kruskal-Wallis test (with multiple subgroups) were used for a preliminary exploration of factors associated with LOS in univariate analysis and to evaluate the distributions of hospitalization costs between subgroups. Multiple linear regression was used to select variables which significantly predicted LOS. To avoid overfitting the model, variables found to be significant (*p* <0.10, inclusion criteria) in the univariate analysis were included.

Data filtering was performed using Excel and analyses were carried out using IBM SPSS Statistics 20. *P* < 0.05 was considered to be statistically significant.

## Results

### Demographic, clinic characteristics of the study population

A total of 1528 patients were included in the study. Of them, 944 were men (61.78%), with a mean age of 61.78 years (median: 60 years), and mean LOS of 11.65 days (median: 10 days). Urban Employee Basic Medical Insurance, Urban Resident Basic Medical Insurance, and the NCMS covered 44.82% (*n* = 685) of patients, and 96.20% (*n* = 1470) patients were admitted as outpatients. Most of the patients were retired (47.19%). Data from Table 1 shows that most inpatients (81.74%) had chronic complications (Table 1).

**Table 1:** Demographic and clinical characteristics of the study population

Characteristics		Mean ± SD or number	Median or percentage	25th–75th percentile
Gender	Male	944	61.78	
	Female	584	38.22	
Age group	20~	16	1.05	
	30~	53	3.47	
	40~	303	19.83	
	50~	365	23.89	
	60~	268	17.54	
	70~	287	18.78	
	80~	218	14.27	
	90~	18	1.18	
LOS		11.65 ± 6.13	10	8–15
Type of medical insurance	Urban Resident Basic Medical Insurance	41	2.68	
	Urban Employee Basic Medical Insurance	603	39.46	
	Other means of payment	23	1.51	
	Public expense	31	2.03	
	Private expense	789	51.64	
	NCMS	41	2.68	
Mode of Admission	Emergency	29	1.90	
	Outpatient	1470	96.20	
	Other	29	1.90	
Year of admission	2013	724	47.38	
	2014	804	52.62	
Occupation	Employee	248	16.23	
	Student	3	0.20	
	Labourer	181	11.84	
	Retired	721	47.19	
	Unemployed	375	24.54	
Complications	No complications	164	10.73	
	Chronic complications	1249	81.74	
	Acute + chronic complications	115	7.53	
Costs per patient		232.47 ± 164.95	185.00	142.04–272.18

### Univariate analysis result

The Mann–Whitney *U* test (with 2 subgroups) and Kruskal-Wallis test (with multiple subgroups) showed that no significant difference was found between LOS and gender (*p* =0.056), mode of admission (*p* =0.057), or year of admission (*p* =0.998). Age group, type of medical insurance, occupation, and type of complications (*p <* 0.001) were significantly associated with LOS. Inpatients aged 90 and over, receiving public financial assistance, retired, and without complications had a longer LOS than other members within the group, their mean LOS were 19.94 ± 5.96 days, 14.19 ± 6.11 days, 12.54 ± 6.29 days, 15.43 ± 7.17 days, respectively (Table 2).

**Table 2:** Factors associated with LOS by univariate analysis

Factors		Mean days	Median days	*P*-value
Gender	Male	11.40 ± 6.04	10.00 (7.00–14.00)	0.056
	Female	12.04 ± 6.27	11.00 (8.00–15.00)	
Age group	20~	9.06 ± 3.79	9.00 (6.00–12.75)	<0.001
	30~	10.00 ± 5.63	9.00 (7.00–10.50)	
	40~	10.95 ± 6.21	10.00 (7.00–13.00)	
	50~	9.83 ± 5.09	9.00 (7.00–13.00)	
	60~	10.71 ± 5.12	10.00 (8.00–13.00)	
	70~	12.45 ± 5.99	11.00 (9.00–15.00)	
	80~	15.67 ± 6.73	16.00 (11.00–21.00)	
	90~	19.94 ± 5.96	21.00 (16.75–23.25)	
Type of medical insurance	Urban Resident Basic Medical Insurance	10.31 ± 5.39	9.00 (7.00–11.00)	<0.001
	Urban Employee Basic Medical Insurance	10.14 ± 4.84	9.00 (7.00–12.00)	
	Other	13.30 ± 5.41	13.00 (11.00–17.00)	
	Public expense	14.19 ± 6.11	15.00 (10.00–19.00)	
	Private expense	12.65 ± 6.74	12.00 (8.00–17.00)	
	NCMS	12.95 ± 7.15	11.00 (8.50–15.50)	
Occupation	Employee	10.95 ± 6.20	10.00 (7.00–15.00)	<0.001
	Student	12.00 ± 5.57	13.00 (6.00–)	
	Labourer	10.32 ± 5.26	9.00 (7.00–13.00)	
	Employed	12.54 ± 6.29	11.00 (8.00–16.00)	
	Unemployed	11.02 ± 5.94	10.00 (8.00–13.00)	
Mode of Admission	Emergency	10.62 ± 7.81	9.00 (4.50–13.00)	0.057
	Outpatient	11.68 ± 5.95	10.00 (8.00–15.00)	
	Other	11.03 ± 11.33	9.00 (2.00–14.50)	
Year of admission	2013	11.67 ± 6.31	10.00 (8.00–14.00)	0.998
	2014	11.62 ± 5.97	10.00 (8.00–15.00)	
Complications	No complications	15.43 ± 7.17	15.50 (10.00–22.00)	<0.001
	Chronic complications	11.23 ± 5.84	10.00 (8.00–14.00)	
	Acute + chronic complications	10.74 ± 5.81	10.00 (7.00–13.00)	

### Multiple linear regression analysis

Age group, type of medical insurance, occupation, and type of complications which were found to be statistically significant in the univariate analysis were predictors, and the LOS was the outcome variable for analysis. According to the multiple linear regression analysis of LOS, it could be concluded that age group, type of medical insurance, and complications were the factors associated with LOS. Inpatients with NCMS had a longer LOS than with Urban Resident Basic Medical Insurance. Compared with inpatients aged 20–30 years, those aged 80–90 had a longer LOS. Moreover, inpatients aged 90 and over had the longest LOS. A significant difference was found between LOS and complications as well. Compared to inpatients without complications, inpatients with chronic complications and with acute + chronic complications had longer LOS (Table 3).

**Table 3:** Factors associated with LOS by multiple linear regression analysis

Factors	Reference group	*ß*	Std. Error	95% C.I		*P*-value
				Lower	Upper	
Gender						
Female	Male	0.165	0.319	−0.459	0.790	0.604
Age group						
30~	20~	1.339	1.761	−2.112	4.790	0.447
40~		2.091	1.612	−1.069	5.252	0.195
50~		0.591	1.616	−2.577	3.758	0.715
60~		1.325	1.642	−1.893	4.543	0.420
70~		2.856	1.645	−0.368	6.081	0.083
80~		5.161	1.671	1.887	8.435	0.002
90~		8.328	2.157	4.101	12.555	0.001
Type of medical insurance						
Urban Employee Basic Medical Insurance	Urban Resident Basic Medical Insurance	−0.050	0.921	−1.856	1.756	0.957
Other		2.541	1.486	−0.372	5.454	0.087
Public expense		2.250	1.383	−0.460	4.961	0.104
Private expense		1.489	0.919	−0.311	3.290	0.105
NCMS		3.053	1.265	0.574	5.531	0.016
Occupation						
Labourer	Employee	0.118	0.577	−1.014	1.249	0.839
Retired		0.843	0.483	−0.104	1.790	0.081
Unemployed		0.506	0.471	−0.416	1.429	0.282
Mode of Admission						
Outpatient	Emergency	1.331	1.079	−0.784	3.445	0.217
Other		0.348	1.507	−2.605	3.301	0.817
Complications						
Chronic complications	No complications	−1.678	0.548	−2.751	−0.605	0.002
Acute + chronic complications		−2.475	0.753	−3.951	−0.998	0.001

The occupation “student” was not included in the multiple linear regression analysis because the sample size was limited to 3

### Cost differencess between various complications

The mean cost per patient was US$232.47, and the total costs were US$159.72 ± 130.83, US$240.60 ± 166.58, and US$247.98 ± 166.22 for inpatients with no complications, chronic complications, and acute + chronic complications, respectively, as shown in Table 4. The distribution of costs for total expenses, drugs, accommodation, imaging, laboratory analysis, diagnostics, and nursing care were significantly different not only between inpatients without complications and those with chronic complications but also between inpatients without complications and those with acute + chronic complications (*p* < 0.001). The distribution of costs for therapeutics and sanitary materials were significantly different between the three different types of complications (*p* < 0.001). We can conclude inpatients without complications spent less than those with other types of complications on total costs and on the various individual expenses. Drugs and laboratory analysis were found to be the greatest expenses, and the least was nursing care (Table 4).

**Table 4:** Hospitalization costs of type 2 diabetes in patients with various complications

Hospitalization costs	No complications		Chronic complications		Acute + chronic complications		*P*-value
	Mean ± SD	Median	Mean ± SD	Median	Mean ± SD	Median	
Total costs	159.72 ± 130.83	135.33 (92.03–184.28)	240.60 ± 166.58	192.09 (147.81–282.30)^a^	247.98 ± 166.22	200.99 (155.47–286.39)^b^	<0.001
Drugs	68.69 ± 75.18	57.84 (27.98–84.92)	111.60 ± 84.44	89.60 (61.67–138.62)^a^	115.59 ± 82.89	95.45 (62.99–135.40)^b^	<0.001
Accommodation	10.28 ± 12.35	7.71 (4.24–12.95)	14.90 ± 19.19	11.64 (8.81–15.80)^a^	13.28 ± 6.99	11.11 (8.81–16.26)^b^	<0.001
Imaging	16.48 ± 14.61	15.03 (4.98–23.06)	25.65 ± 19.87	22.35 (13.26–33.55)^a^	23.93 ± 17.13	22.06 (11.15–31.75)^b^	<0.001
Therapeutics	14.41 ± 36.90	6.63 (2.32–11.38)	27.12 ± 48.69	10.92 (5.79–20.07)^a^	31.15 ± 49.19	13.84 (8.48–24.89)^bc^	<0.001
Lab analysis	32.85 ± 22.34	31.55 (19.01–46.50)	44.62 ± 26.04	39.72 (28.33–56.05)^a^	44.44 ± 25.33	38.76 (25.68–58.61)^b^	<0.001
Sanitary materials	5.25 ± 5.69	3.78 (1.84–6.81)	11.48 ± 62.34	5.31 (3.35–7.77)^a^	14.31 ± 32.36	6.54 (4.31–9.76)^bc^	<0.001
Diagnostics	1.55 ± 1.08	1.42 (0.75–2.06)	2.24 ± 1.06	2.06 (1.53–2.68)^a^	2.27 ± 1.01	2.07 (1.53–2.87)^b^	<0.001
Nursing care	1.09 ± 1.77	0.76 (0.47–1.32)	1.65 ± 1.45	1.15 (0.86–1.91)^a^	1.82 ± 1.55	1.23 (0.85–2.10)^b^	<0.001

A Dunn’s nonparametric comparison for post hoc testing after a Kruskal-Wallis test were performed and indicated significant differences between groups of no complications and chronic complications, no complications and acute + chronic complications, and chronic complications and acute + chronic complications, as illustrated a, b, c respectively in Table 4

### Cost differences between various chronic complications

Chronic complications in type 2 diabetes patients was divided into three categories: microvascular, macrovascular, and microvascular + macrovascular complications as shown in Table 5. The total costs were US$225.40 ± 115.32, US$221.25 ± 177.64, and US$ 275.18 ± 193.14, respectively. The distribution of total costs and accommodation costs were significantly different between the three categories (*p* < 0.001). The distribution of costs for therapeutics and nursing care were significantly different not only between inpatients with microvascular complications and with microvascular + macrovascular complications but also between inpatients with macrovascular complications and with microvascular + macrovascular complications (*p* < 0.001). The distribution of costs for drugs, imaging, laboratory analysis, sanitary materials, and diagnostics showed significant differences between inpatients with macrovascular complications and with microvascular complications as well as between inpatients with macrovascular complications and with microvascular + macrovascular complications (*p* < 0.001). From the perspective of the total costs, inpatients with microvascular + macrovascular complications spent the most. Drugs and laboratory analysis were the highest costs and nursing care was the lowest (Table 5).

**Table 5:** Hospitalization costs of type 2 diabetes in patients with various chronic complications

Hospitalization costs	Microvascular		Macrovascular		Microvascular + macrovascular		*P*-value
	Mean ± SD	Median	Mean ± SD	Median	Mean ± SD	Median	
Total costs	225.40 ± 115.32	200.34 (151.00–263.24)	221.25 ± 177.64	170.05 (134.81–255.11)^a^	275.18 ± 193.14	217.91 (158.94–345.02)^bc^	<0.001
Drugs	109.11 ± 67.82	94.80 (65.25–137.20)	99.60 ± 76.64	80.26 (57.07–119.78)^a^	125.87 ± 103.22	96.08 (63.04–164.70)^c^	<0.001
Accommodation	13.12 ± 7.55	11.72 (8.99–15.09)	13.24 ± 13.98	10.11 (7.71–15.42)^a^	18.37 ± 29.02	12.38 (9.91–17.73)^bc^	<0.001
Imaging	28.19 ± 19.55	24.26 (15.97–35.40)	21.84 ± 16.31	19.85 (10.84–29.24)^a^	26.74 ± 22.65	22.61 (11.48–35.99)^c^	<0.001
Therapeutics	16.08 ± 20.87	10.76 (5.71–16.61)	29.55 ± 54.91	10.33 (5.31–19.92)	36.14 ± 59.49	11.71 (6.47–29.3)^bc^	0.005
Lab analysis	45.96 ± 25.19	40.30 (28.55–58.62)	40.44 ± 19.78	37.33 (27.69–50.51)^a^	47.30 ± 31.32	41.37 (28.94–60.36)^c^	0.007
Sanitary materials	7.14 ± 10.02	5.52 (3.69–7.46)	12.15 ± 95.75	4.64 (2.84–7.01)^a^	15.31 ± 49.03	6.02 (3.59–9.15)^c^	<0.001
Diagnostics	2.22 ± 0.95	2.10 (1.69–2.62)	2.08 ± 0.99	1.87 (1.50–2.62)^a^	2.40 ± 1.21	2.25 (1.69–2.87)^c^	<0.001
Nursing care	1.34 ± 1.17	1.12 (0.85–1.42)	1.55 ± 1.36	1.05 (0.76–1.87)	2.08 ± 1.69	1.63 (0.96–2.62)^bc^	<0.001

A Dunn’s nonparametric comparison for post hoc testing after a Kruskal-Wallis test were performed and indicated significant differences between groups of microvascular complications and macrovascular complications, microvascular complications and microvascular + macrovascular complications, and macrovascular complications and microvascular + macrovascular complications, as illustrated a, b, c respectively in Table 5

## Discussion

### LOS and demographic and clinical characteristics

Through this study, Wuhan, a medium-sized city in China where LOS of inpatients with diabetes (means: 11.65 ± 6.13 days; median: 10.00 days) were slightly shorter than those reported in Shantou, China (means: 13.91 days; median: 10.00 days) [15] but slightly longer than those found abroad (means: 8.2 days) [12]. These discrepancies may stem from the type and grade of hospital, the standard charges, the conditions for care, and the social and cultural environment [16, 17].

The findings of the multiple linear regression analysis were unlike much previous research [18, 19] in that people with the new cooperative medical scheme had a longer LOS than people with Urban Resident Basic Medical Insurance and with Urban Employee Basic Medical Insurance. Despite a relatively high coverage of medical insurance in China, the coverage provided by different types of social medical insurance in the country varies greatly. At present, Urban Employee Basic Medical Insurance, as an earlier developed and more mature social medical insurance in China, is superior to other social medical insurance plans in its coverage and cost in terms of deductible and rate of reimbursement. The difference mentioned above is the main reason why Urban Employee Basic Medical Insurance would increase the cost of hospitalization and the LOS. However, our study draws different conclusions, which can be explained as follows: Zhongnan Hospital, as a large tertiary general hospital, serves not only the immediate urban area but also the rural area which surrounds it. The current grading clinics allow patients living in rural areas, most of whom are enrolled in the new cooperative medical schemes, to come to Zhongnan Hospital for further treatment of more serious conditions. Thus, the results of this study showed that the LOS of patients with the new cooperative medical scheme were longer. From these results, we can conclude that establishing an unified basic medical insurance system for urban and rural residents to guarantee basic medical insurance rights and interests, promote social fairness, and encourage urban–rural coordinated development is needed. China is currently exploring a new way to integrate the medical insurance system of urban and rural areas [20].

In addition, the multiple linear regression analysis showed another result different from many studies which indicated that people without complications had a longer LOS than people with chronic complications and acute + chronic complications [21–23]. The mean LOS were 11.23 ± 5.84 and 10.74 ± 5.81 days respectively for patients with chronic complications and acute + chronic complications as opposed to 15.43 ± 7.17 days for patients without complications. One possible reason for this is that most inpatients without complications were hospitalized for the first time, or this was the first time the disease had been diagnosed, so they paid more attention to the disease and accepted longer hospitalization in order to control the development of the disease. However, patients with complications may have been more likely to receive a shorter hospitalization and then maintain drug treatment at home.

### Costs and various type of complications

The result of the study showed that the average per patient cost of diabetes was US$232.47 and the median cost was US$185.00. This is different from that found in some other parts of China and abroad [24, 25]. For instance, the average cost per patient was far less than that in Europe and the United Arab Emirates [11], but was slightly higher than the US$152 per patient in Iran [26]. China is a developing country, with its own unique national conditions, medical and health service systems, hospital grades and settings, and these may be responsible for the discrepancy in average per patient cost. According to a survey of resident income in China, GDP per capita was US$4410.59 (29,992 RMB, with an exchange rate for US$ to RMB of 1:6.8 in 2010) [27]. The average hospitalization cost for diabetic patients was 5.27% of the GDP per capita and 55.35% of the per capital expenditure for health care (http://data.worldbank.org.cn/indicator/SH.XPD.PCAP/countries). This means diabetes is a huge burden both for residents and for the health care system. Other studies have come to the same conclusion [28].

This study found that costs for inpatients with complications were higher than for patients without complications, and numerous studies have drawn similar conclusions [29, 30]. This study also found total costs for patients with microvascular + macrovascular complications were higher than those for patients with microvascular complications alone. Moreover, this research showed that patients with macrovascular complications spent the least. These findings which were completely contrary to the conclusions of the study conducted in Europe and the United Arab Emirates, which found that patients with microvascular + macrovascular complications spend the most, followed by those with macrovascular complications, in that study, patients with microvascular complications spent the least [11]. As the disease progresses, a series of complications will inevitably occur, either with regard to personal health or in terms of the social economic burden, preventing and controlling the complications of diabetes, especially microvascular complications, are essential.

Regardless of the kind of complication, drugs were the highest type of cost and nursing care the least. Therefore, in order to reduce the burden of the disease in patients with type 2 diabetes, measures should be taken to reduce drug costs in conjunction with the implementation of drug price reform policies in Hubei Province. Moreover, because diabetes is a metabolic disease, in addition to access to affordable medication, health education and lifestyle interventions are also very important, as many studies have shown [31, 32]. In this way, both LOS and hospital costs would decrease.

### Limitations

Because this study was conducted with data from a single hospital, findings might have been different in other parts of the country or in other types of hospitals. In addition, the data was limited to the period between April 1, 2013, and March 31, 2014, a relatively short time span; thus, a continuous trend comparison could not be made. Also, for patients undergoing two or more hospitalizations, each hospitalization was studied as a separate case, which may have led to an underestimate of hospital costs. Moreover, some cardiovascular diseases are not limited to patients with diabetes but may occur before diabetes, so some of the costs might not have been caused by diabetes, leading to an overestimation of hospital costs. Finally, the data for demographic and clinic characteristics were limited (e.g., the results of laboratory tests), if more data for demographic and clinical characteristics had been available, this research would be more substantial.

## Conclusions

The LOS for patients with diabetes in China were found to be longer than those in foreign countries. It should be appropriate to take measures, such as integrating the medical insurance system of urban and rural areas as well as monitoring and educating patients at home. Overall, the costs for patients with complications were higher than for patients without complications, so preventing and controlling the complications of diabetes is imperative, especially microvascular complications.

## Additional file

Additional file 1: LOS and direct costs of type2 diabetes. Title of data: Sheet1 (LOS and direct costs of all type2 diabetes patients); Sheet2 (LOS and direct costs of type2 diabetes patients with chronic complications). Description of data: Basic information such as type of medical insurance, gender, age, occupation, method of admission, LOS, type of complications and cost information for drugs, accommodation, imaging, therapeutics, laboratory analysis, sanitary materials, diagnostics, and nursing care are included. (XLSX 540 kb)

## Abbreviations

$: US dollars
GDP: Gross Domestic Product
HIS: Hospital Information System
LOS: Length of stay
NCMS: New Cooperative Medical Scheme
RMB: Ren Min Bi
SD: Standard deviation
